# Reduced chemodiversity suppresses rhizosphere microbiome functioning in the mono-cropped agroecosystems

**DOI:** 10.1186/s40168-022-01287-y

**Published:** 2022-07-16

**Authors:** Pengfa Li, Jia Liu, Muhammad Saleem, Guilong Li, Lu Luan, Meng Wu, Zhongpei Li

**Affiliations:** 1 State Key Laboratory of Soil and Sustainable Agriculture, Institute of Soil Science, Chinese Academy of Sciences, Nanjing, 210008 China; 2 Department of Microbiology, Key Lab of Microbiology for Agricultural Environment, Ministry of Agriculture, College of Life Sciences, Nanjing Agricultural University, Nanjing, 210095 China; 3 Soil and Fertilizer & Resources and Environment Institute, Jiangxi Academy of Agricultural Sciences, Nanchang, 330200 China; 4 Department of Biological Sciences, Alabama State University, Montgomery, AL 36104 USA

**Keywords:** Rhizodeposits, Chemodiversity, DNA-SIP, Microbiome functioning, Mono-cropped agroecosystem

## Abstract

**Background:**

Rhizodeposits regulate rhizosphere interactions, processes, nutrient and energy flow, and plant-microbe communication and thus play a vital role in maintaining soil and plant health. However, it remains unclear whether and how alteration in belowground carbon allocation and chemodiversity of rhizodeposits influences microbiome functioning in the rhizosphere ecosystems. To address this research gap, we investigated the relationship of rhizosphere carbon allocation and chemodiversity with microbiome biodiversity and functioning during peanut (*Arachis hypogaea*) continuous mono-cropping. After continuously labeling plants with ^13^CO_2_, we studied the chemodiversity and composition of rhizodeposits, along with the composition and diversity of active rhizosphere microbiome using metabolomic, amplicon, and shotgun metagenomic sequencing approaches based on DNA stable-isotope probing (DNA-SIP).

**Results:**

Our results indicated that enrichment and depletion of rhizodeposits and active microbial taxa varied across plant growth stages and cropping durations. Specifically, a gradual decrease in the rhizosphere carbon allocation, chemodiversity, biodiversity and abundance of plant-beneficial taxa (such as *Gemmatimonas*, *Streptomyces*, *Ramlibacter*, and *Lysobacter*), and functional gene pathways (such as quorum sensing and biosynthesis of antibiotics) was observed with years of mono-cropping. We detected significant and strong correlations between rhizodeposits and rhizosphere microbiome biodiversity and functioning, though these were regulated by different ecological processes. For instance, rhizodeposits and active bacterial communities were mainly governed by deterministic and stochastic processes, respectively. Overall, the reduction in carbon deposition and chemodiversity during peanut continuous mono-cropping tended to suppress microbial biodiversity and its functions in the rhizosphere ecosystem.

**Conclusions:**

Our results, for the first time, provide the evidence underlying the mechanism of rhizosphere microbiome malfunctioning in mono-cropped systems. Our study opens new avenues to deeply disentangle the complex plant-microbe interactions from the perspective of rhizodeposits chemodiversity and composition and will serve to guide future microbiome research for improving the functioning and services of soil ecosystems.

Video abstract

**Supplementary Information:**

The online version contains supplementary material available at 10.1186/s40168-022-01287-y.

## Background

The rhizosphere is the interface between plant roots and soil where interactions among numerous microbes determine biogeochemical cycling, plant growth, and tolerance to biotic and abiotic stresses [1–6]. Plants exude a large proportion of photosynthetically fixed carbon (C) into the rhizosphere through their roots, and these organic compounds are collectively named as rhizodeposits [7]. The rhizodeposits act as vinculums to link plants with soil microbes and nutrients, thus regulating a wide range of biological functions and ecosystem processes such as nutrient mobilization, cycling, availability, and sequestration [8, 9]. Because of their multifunctional properties, rhizodeposits, nevertheless, fuel rhizosphere microbial communities, interactions, and functions that are important for plant growth and development [10–12]. However, the rhizosphere processes can hardly be tracked because it is considered to be one of the most dynamic interfaces on Earth, thus making it difficult to deeply understand the functional ecology of the rhizosphere ecosystem [13]. This calls for further research on rhizodeposits, their chemistry, chemodiversity, and dependent microbes to mechanistically understand their ecophysiological functions in the rhizosphere ecosystem.

The rhizodeposition is mainly influenced by several soil- and plant-derived factors such as edaphic properties, plant species, performance, growth stage, and other environmental interactions [14, 15]. In agroecosystems, the C allocation belowground is, therefore, highly influenced by field management practices such as monoculture and intercropping, which ultimately shape soil properties and plant performance over time [16]. Under long-term monoculture cropping, the alterations in soil nutrient contents (e.g., accumulation of nitrogen-species) may suppress belowground C allocation by plants according to the resource optimization hypothesis [17, 18]. Furthermore, long-term monoculture cropping often suppresses plant growth [19], which may also have the potential to negatively affect the belowground C allocation though not empirically tested before rigorously [20, 21]. Given the impact of agricultural practices on soil properties and plant performance [22–24], we expect that continuous mono-cropping may influence belowground C allocation, chemodiversity, and composition of rhizodeposits.

Different amounts and types of rhizodeposits may select for different rhizosphere microbial communities [4, 25, 26]. If continuous mono-cropping does influence rhizodeposits, we would expect that alterations in rhizodeposits would consequently affect the rhizosphere microbiomes including their diversity, composition, and community assembly mechanisms (determinism-stochasticity balance), especially of the active microbial taxa that depend on rhizodeposits for nutrient and energy requirements. However, this aspect has never been tested in earlier studies. The microbial functions, from an individual cell to community level responses, are highly determined by the environmental conditions such as temperature, pH, and resource availability [27–29]. Recent studies have predicted the importance of environmental metabolites, their composition, and chemodiversity in structuring microbial communities and steering their functions in the aquatic and soil ecosystems [30–32]. However, the exact relationship between habitat chemodiversity and microbial functions in dynamic ecosystems, such as rhizosphere, remains unclear. The chemodiversity of rhizosphere ecosystems may dramatically change with crop growth cycle, cropping years, and agricultural practices, though not empirically proved yet. Therefore, whether and how alternation in chemodiversity influences microbial functions in the rhizosphere requires well-replicated and time-series studies. Meanwhile, the continuous mono-cropping systems provide an ideal opportunity to elucidate the role of chemodiversity in the functioning of rhizosphere microbiome. Given the expected reduction in the chemodiversity of rhizodeposits under continuous mono-cropping, one would also expect reduction or suppression in microbial functions in the rhizosphere. Therefore, we hypothesize that continuous mono-cropping will reduce the chemodiversity of rhizodeposits, and resultantly, it will suppress the biodiversity and functions of microbial communities that actively utilize rhizodeposits.

**Fig. 1:**
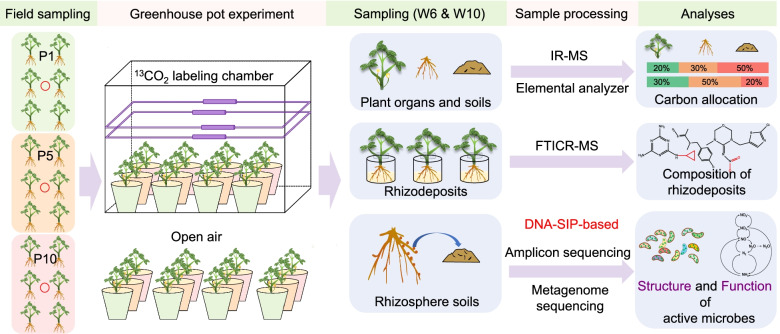
Flow diagram of the key experimental settings in the current study. *Field sampling*: peanut plants were grown in soils collected from fields representing different years of continuous monoculture cropping. Specifically, P1, P5, and P10 represent soils under continuous monoculture cropping of peanut for 1, 5, and 10 years, respectively. Soils were collected in April 2019 for greenhouse pot experiment. *Greenhouse experiment*: on 10 May 2019, each experimental pot was filled with 3 kg of the soil, and three surface-disinfect peanut seeds were sown in it. On 2 June 2019, four biological replicate pots of each treatment were randomly selected and were then uniformly labeled with ^13^CO2 in a temperature-controlled glass chamber. Four other biological replicate pots were cultivated in open air as control. On 2 July 2019, another four biological replicate pots of each treatment were randomly selected and were then uniformly labeled with ^13^CO2. Similarly, four other biological replicate pots were also cultivated in open air as control. *Sampling*: on 23 June 2019 (W6: about 6 weeks after seeds were sown)/23 July 2019 (W10: about 10 weeks after seeds were sown), we stopped the labeling. Then, the plant organs, soils, rhizodeposits, and rhizosphere soils were collected. Samples were then processed for subsequent analyses

Here, we tested our hypothesis by conducting a greenhouse experiment in the peanut (*Arachis hypogaea*) continuous monoculture system (Fig. 1). Specifically, we investigated how continuous peanut monoculture affects (i) the belowground C allocation, chemodiversity, and composition of rhizodeposits; (ii) the biodiversity, composition, and assembly of microbial communities that actively utilize rhizodeposits; and (iii) consequently, the functional potentials of rhizosphere active microbes.

## Materials and methods

### Field experimental setup

The field experiment was conducted in the Comprehensive Experimental Station of red soil (Dongxiang County, Jiangxi Province, 28° 10′ 59″ N, 106° 35′ 11″ E). The experimental soil was classified as Udic Ferroso [33], which is commonly known as red soil in China. The mean annual precipitation and temperature are 2180.6 mm and 18 °C, respectively. The soil parent material, field terrain, cropping, and management history of the experimental fields were consistent before conducting the mono-cropping experiment, while all experimental fields were adjacent (< 50 m). Peanut plants were grown in soils collected from fields representing different years of continuous monoculture cropping. Specifically, P1, P5, and P10 represented soils under continuous monoculture cropping of peanut for 1, 5, and 10 years, respectively. Each treatment has three real field replicates (plots), and each field plot is 4 m × 10 m. Soils were collected in April 2019 for pot experiment. The experimental setup is summarized in Fig. 1. For each treatment, multiple soil samples were collected from experimental field plots, and then combined and homogenized, and their physicochemical properties are given (Table S1).

### Pot experiment, continuous ^13^C labeling, and sample collection

On 10 May 2019, each experimental pot was filled with 3 kg of the soil, and three surface-disinfect peanut (Ganhua-5) seeds were sown in it. Hence, for each treatment, there were sixteen biological replicate pots, for a total of 48 experimental units. On 19 May 2019, two seedlings in each pot were removed, and only one seedling in each pot was maintained to ensure consistent growth and density of peanut plants per pot. On 2 June 2019, four biological replicate pots of each treatment were randomly selected and were then uniformly labeled with ^13^CO_2_ in a temperature-controlled glass chamber (100 × 80 × 100 cm height). The chamber was placed in the greenhouse to expose it to the natural sunlight. ^13^CO_2_ was released by Na_2_^13^CO_3_ (99 atom% ^13^C, 1 M) with H_2_SO_4_ (1 M). Constant CO_2_ concentration (450 μL CO_2_ L^−1^) inside the chamber was achieved via automatic further reactions. The chamber temperature was maintained at < 36 °C by activating air-conditioning via the data-logger script. On 23 June 2019 (W6: flowering stage, about 6 weeks after seeds were sown), we stopped the labeling. Then, the rhizosphere soils were very gently collected and stored at −40 °C until further use within 1 week. After cleaning the roots with ultrapure water, plants were put into a plastic bottle (500 mL, wrapped with tin foil to avoid photodegradation) with 200 mL of ultrapure water to collect rhizodeposits. About 20 h later, plants were removed from the bottles and dried in the bake oven. The solutions in the bottle were stored at 4 °C. The soils in the pot after harvesting the plants were air-dried and homogenized. By doing these, we collected rhizosphere soil, rhizodeposits, and plant samples of the labeled and non-labeled (control) peanut plants. On 2 July 2019, another four biological replicate pots of each treatment were randomly selected and were then uniformly labeled with ^13^CO_2_, and the samples were collected on 23 July 2019 (W10: pegging and podding stage, about 10 weeks after seeds were sown) following the same procedures as described above. In total, 24 (3 treatments × 2 sampling stages × 4 replicates) rhizosphere soil samples, 24 rhizodeposits samples, and 24 plant samples were collected. Correspondingly, the same number of non-labeled samples was collected. The physicochemical properties of rhizosphere soil samples are given (Table S1).

### Determination of C allocation

The dry plants were divided into two parts, namely the shoot and root portions. Then, these plants were grounded into powder and homogenized. The total C and stable C isotope ratio (δ^13^C) contents of plant and soil samples were analyzed using an isotope ratio mass spectrometer, coupled with an elemental analyzer (Thermo-Fisher Scientific, Waltham, MA, USA). The amount of photosynthesized ^13^C accumulated in the plant tissues (shoots and roots) and soils was calculated using the equations described before [34] (Text S1).

### Determination of rhizodeposits composition

The rhizodeposits were extracted from the solution using ethyl acetate on a vacuum rotary evaporator and then were dissolved into methanol. The Fourier-transform ion cyclotron resonance MS (FTICR-MS) was used to analyze the molecular structure of rhizodeposits with high throughput and high resolution. The detailed information about FTICR-MS sample preparation is given (Text S2). Data analysis software (Bruker Daltonics version 4.2) was used to convert raw spectra to final values (m/z) using the FTMS peak picker (S/N threshold of 7; absolute intensity threshold of 100). To reduce cumulative errors, all peaks from the entire dataset were aligned to each other to check the mass shift. The molecular formulae of peaks were calculated using a custom software. Considering competition between charges could cause peak intensities less informative [35], we performed all analyses by using the binary presence or absence values instead of peak intensities [36]. The chemodiversity of rhizodeposits was the number of detected effective peaks in each sample.

### Soil DNA extraction and DNA-SIP experiment

The genomic DNA was extracted from 0.5 g rhizosphere soil by using the FastDNA SPIN Kit for soil (MP Biomedicals, Santa Ana, CA). The isopycnic density gradient centrifugation with 3.0 μg of extracted DNA was performed as previously described [37]. The gradient centrifugation yielded fifteen DNA fractions (approximately 340 μL each) for each sample, and the abundance of prokaryotic 16S rRNA genes from each fraction isolated by the gradient centrifugation was quantified by quantitative PCR on a CFX96 Optical Real-Time Detection System (Bio-Rad, Hercules, CA, USA). The amplification efficiencies were 88.9–100%, with *R*^2^ values of 0.979–1.000. The detailed information regarding isopycnic density gradient centrifugation and quantitative PCR is given (Text S3, Fig. S1, Table S2). The DNA in labeled heavy fractions was considered to be derived from active microbes that can utilize the rhizodeposits.

### Amplicon high-throughput sequencing and data processing

The PCR amplification of the ^13^C-DNA of the heavy fractions was conducted for bacterial community analysis using 519F and 907R primers [38] (Table S3). We performed high-throughput sequencing using the Illumina MiSeq sequencing platform (Illumina Inc., CA, USA). The detailed information about the processing of sequencing data including raw sequences trimming, amplicon sequence variants (ASV) clustering, taxonomic assignment, sequence rarefaction, and phylogenetic dendrogram construction is provided in Supplementary information (Text S4).

### Metagenome sequencing and data processing

The ^13^C-DNA of the heavy fractions from the DNA-SIP experiment was used to construct libraries for metagenomic sequencing on the Illumina HiSeq 4000 platform (Illumina Inc., San Diego, CA, USA). As we failed to construct clone libraries for many samples in W6, only samples in W10 were sequenced and analyzed. This yielded 94.67 Gb of raw data and 91.18 Gb of clean data after quality control (Table S4). The assembly of metagenomes and protein-coding genes was performed as described previously [39]. All genes in the catalogue were translated to amino acid sequences and aligned with data in the Kyoto Encyclopedia of Genes and Genomes (KEGG) database. Each protein was assigned a KEGG ortholog (KO) based on the best-hit gene in the KEGG database. The current study yielded 7806 KOs and 409 KEGG pathways. The detailed information about metagenome sequencing and analysis is described in the Supplementary information (Text S5).

### Data analysis

Significant difference between treatments was determined using R packages *car* and *vegan*. Spearman’s correlation coefficient and significance were calculated using the *rcorr* function in the R package *Hmisc*. The random forest model was conducted using R packages *randomForest* and *rfPermute* to quantitatively illustrate the important predictors of microbial biodiversity. Three relational dendrograms (MCD: based on molecular characteristics; TD: based on potential biochemical transformations; and TWCD: based on transformation-weighted characteristics) of rhizodeposits were constructed following the method described previously [36] (Fig. S2, Text S6). We then investigated the assembly processes of rhizodeposits and active microbial communities using the phylogeny-informed models [40]. Detailed methods about the data analyses model are given (Text S6).

## Results

### Plant biomass and allocation of photosynthesized C

**Fig. 2:**
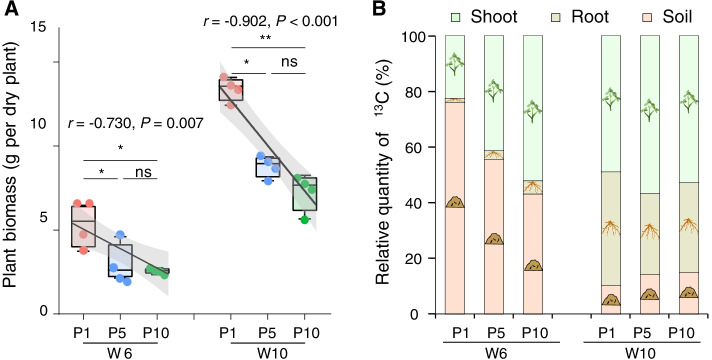
Plant biomass and photosynthesized carbon allocation. **A** The plant biomass at W6 and W10. Lines represent the least squares regression fits, and shaded areas represent the 95% confidence intervals. We applied one-side *F* and two-side *t*-tests and then calculated *P* values as shown. **B** The allocation of photosynthesized carbon at W6 and W10. * and ** indicate significant difference among treatments at *P* < 0.05 and *P* < 0.01, respectively. “ns” represents nonsignificant difference. P1, P5, and P10 represent samples under continuous monoculture cropping of peanut for 1, 5, and 10 years, respectively; W6 and W10 represent samples collected 6 and 10 weeks after sowing, respectively

The plant biomass of P1 was significantly higher than that of P5 and P10 at both growth stages (*P* < 0.05, Fig. 2A), while there was no significant difference between P5 and P10 (*P* > 0.05). At both growth stages, the plant biomass decreased significantly with the prolonged monoculture duration (W6: *r* = −0.730, *P* < 0.05; W10: *r* = −0.902, *P* < 0.05). The allocation of photosynthesized C was highly different between either growth stages or treatments (Fig. 2B). The two-way ANOVA showed that both growth stage (*F*_1.18_ = 20.960, *P* < 0.001) and soil-use history (mono-cropped duration) (*F*_2.18_ = 10.910, *P* < 0.001) had significant effects on the photosynthesized C allocation with growth stage playing a bigger role (Table S5). At W6, peanut plants in P1, P5, and P10 treatments allocated 76, 56, and 43% photosynthesized C into the soil, respectively. At W10, peanut plants in P1, P5, and P10 treatments allocated 10, 14, and 15% photosynthesized C into the soil, respectively. The plant biomass especially belowground plant biomass was significantly positively correlated with belowground C allocation (Table S6). At W6, the soil pH and available phosphorus (AP) were significantly positive, while soil organic carbon (SOC), total nitrogen (TN), available nitrogen (AN), and available potassium (AK) were significantly negatively correlated with belowground C allocation. At W10, of all soil physicochemical properties, only total potassium (TK) showed a significant negative correlation with belowground C allocation (Table S6).

### Composition and chemodiversity of rhizodeposits

**Fig. 3:**
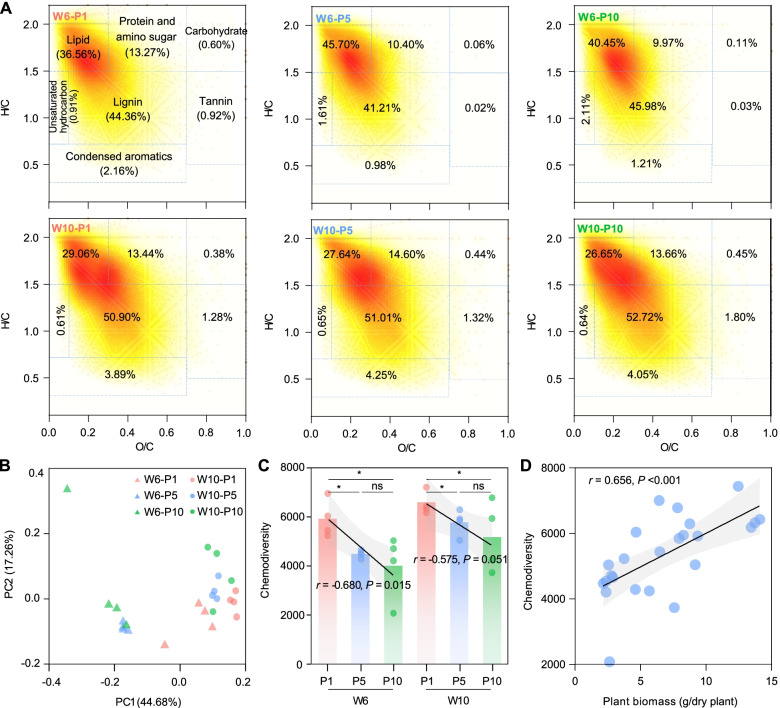
Composition and chemodiversity of rhizodeposits. **A** van Krevelen diagrams from the FTICR-MS spectra of rhizodeposits. **B** Principal coordinate analysis on the composition of peanut rhizodeposits. **C** The chemodiversity of rhizodeposits in different treatments at different growth stages. **D** Correlation between plant biomass and chemodiversity of peanut rhizodeposits. Lines represent the least squares regression fits, and shaded areas represent the 95% confidence intervals. We applied one-side *F* and two-side *t*-tests and then calculated *P*-values as shown. P1, P5, and P10 represent samples under continuous monoculture cropping of peanut for 1, 5, and 10 years, respectively; W6 and W10 represent samples collected 6 and 10 weeks after sowing, respectively

Based on the van Krevelen diagrams (Fig. 3A), the unique molecular formulae determined by the FTICR-MS were used to evaluate the composition and chemodiversity of rhizodeposits. Based on the relative abundance, the lignins, lipids, proteins, and amino sugars were highly dominant in all samples (Fig. S3). The lipids in W10 than W6 were significantly less, while the condensed aromatics showed an opposite tendency. At W6, the condensed aromatics, tannins, and carbohydrates were significantly higher in P1, but the unsaturated hydrocarbons were higher in the P5 and P10 treatments. Except between lignins, proteins, and amino sugars and between lignins and carbohydrates, the relative abundances of all seven categories of rhizodeposits were correlated with each other (Fig. S4).

The principal coordinates analysis based on Sørensen’s distance showed that the composition of rhizodeposits was significantly different between either plant growth stages and or across treatments (Fig. 3B). A two-way PERMANOVA analysis based on Sørensen’s distance indicated that growth stage (*F*_1.18_ = 17.991, *P* <0.001) played a bigger role in affecting the composition of rhizodeposits than year of mono-cropping (*F*_2.18_ = 6.246, *P* < 0.001, Table S8).

The chemodiversity in W10 than W6 was consistently higher; and it tended to significantly decrease with increasing the year of mono-cropping at both growth stages (W6: *r* = −0.680, *P* = 0.015; W10: *r* = −0.575, *P* = 0.051; Fig. 3C). A two-way ANOVA indicated that growth stage (*F*_1.18_ = 7.663, *P* = 0.006) rather than year of mono-cropping (*F*_1.18_ = 6.852, *P* = 0.013) had a greater effect on the chemodiversity of rhizodeposits (Table S5). Additionally, we found a significant positive correlation between the chemodiversity of rhizodeposits and plant biomass (*r* = 0.656, *P* < 0.001, Fig. 3D). We also found that soil pH and AP were significantly positively, while TN was significantly negatively correlated with rhizodeposits chemodiversity at W6; AN was significantly negatively correlated with rhizodeposits chemodiversity at W10 (Table S7).

### Composition and biodiversity of active bacteria

After isopycnic density gradient centrifugation, the DNA in ^13^C-labeled heavy fractions represented the species utilizing the rhizodeposits, and thus, it was further investigated. However, as we failed to sequence some fractions (either heavy or light) of P10, we cannot guarantee the successful labeling and centrifugation of all P10 samples. Therefore, only ^13^C heavy fractions of P1 and P5 were investigated in the downstream analyses.

**Fig. 4.**
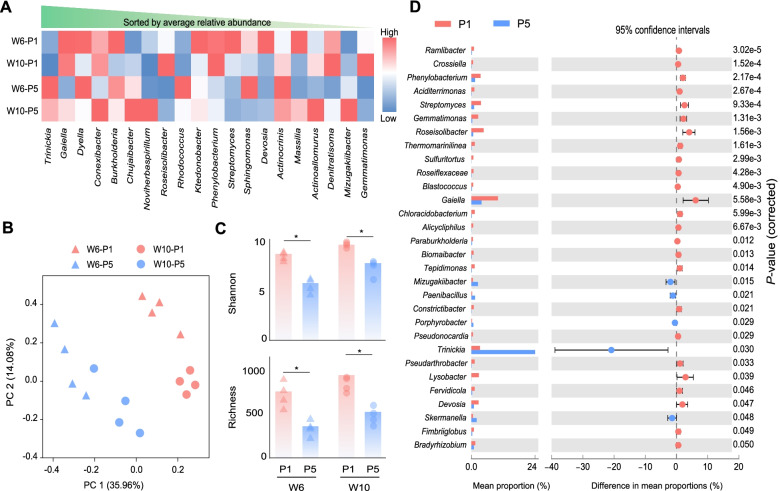
Composition and biodiversity of active bacteria utilizing the rhizodeposits. **A** The normalized relative abundance of top 20 active bacterial genera. **B** PCoA on active bacterial communities. **C** The alpha diversity of active bacteria. **D** The two-tailed Wilcoxon test revealed the abundant differential bacteria in P1 and P5 treatments. P1 and P5 represent samples under continuous monoculture cropping of peanut for 1 and 5 years, respectively; W6 and W10 represent samples collected 6 and 10 weeks after sowing, respectively

Based on the average relative abundance, the active bacteria were highly dominated by *Proteobacteria*, *Actinobacteria*, *Acidobacteria*, *Chloroflexi*, and *Gemmatimonadetes* at phylum level (Fig. S5). At genus level, *Trinickia*, *Gaiella*, *Dyella*, *Conexibacter*, *Burkholderia*, *Chujaibacter*, *Noviherbaspirillum*, *Roseisolibacter*, *Rhodococcus*, and *Ktedonobacter* were top 10 genera utilizing the rhizodeposits (Fig. 4A). The PCoA combined with the PERMANOVA based on the Bray-Curtis dissimilarity index of active bacteria indicated significant differences between either growth stages or mono-cropped years, and the mono-cropped duration (*F*_2.18_ = 8.014, *P* <0 .001) than growth stage (*F*_1.18_ = 3.672, *P* = 0.004) played a bigger role in affecting the composition of active bacteria (Fig. 4B, Table S8).

At both growth stages, the alpha diversity, including Shannon-Wiener index and species richness, of active bacteria in P1 was consistently higher than that of P5, suggesting that more bacterial species were involved in the utilization of rhizodeposits in P1 (Fig. 4C). Moreover, the species richness of active bacteria was significantly positively correlated with belowground C allocation at W6 (*r* = 0.798, *P* = 0.018), but this correlation was weak at W10 (*r* = 0.305, *P* = 0.463, Fig. S6). We then conducted a two-tailed Wilcoxon test to reveal the significantly enriched bacteria in P1 and P5 (Fig. 4D, Fig. S7), and our results showed a total of eighty-three significantly and differentially enriched genera (*P* < 0.05). Among these, 71 were significantly enriched in P1 (such as *Gemmatimonas*, *Streptomyces*, *Ramlibacter*, and *Lysobacter*), while 12 were significantly enriched in P5 such as *Trinickia*.

### Associations between rhizodeposits and active bacteria

**Fig. 5:**
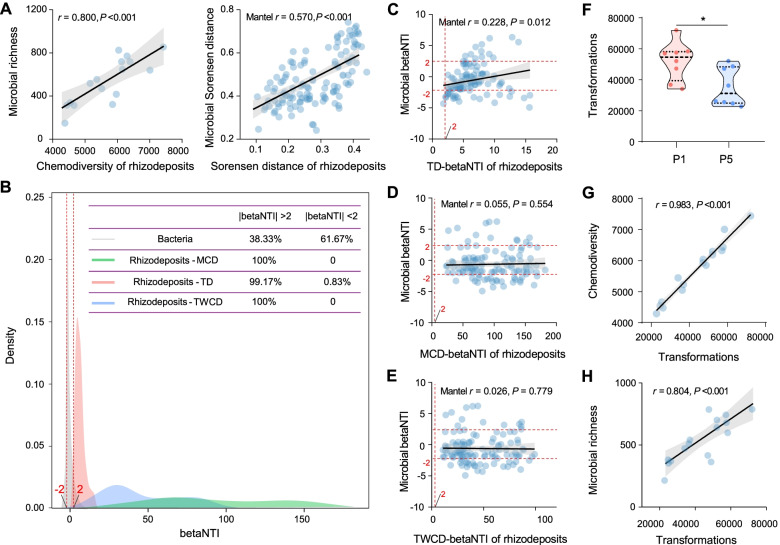
Associations between rhizodeposits and active bacteria. **A** Correlations between rhizodeposits and active bacteria. **B** The distribution of betaNTI values of rhizodeposits and active bacteria. **C–E** The relationships between microbial betaNTI values and TD-based (**C**), MCD-based (**D**), and TWCD-based (**E**) betaNTI values of rhizodeposits. The correlation coefficients were determined by Mantel test. **F** The number of transformations in P1 and P5. *Represents significant difference between P1 and P5 at *P* < 0.05. **G–H** The relationships between number of transformations and chemodiversity of rhizodeposits (**G**) and biodiversity of active bacteria (**H**). Lines represent the least squares regression fits, and shaded areas represent the 95% confidence intervals. We applied one-side *F* and two-side *t*-tests and then calculated *P*-values as shown. P1 and P5 represent samples under continuous monoculture cropping of peanut for 1 and 5 years, respectively

We found a significant correlation between chemodiversity of rhizodeposits and biodiversity of active bacteria (*r* = 0.800, *P* < 0.001, Fig. 5A). Meanwhile, the random forest model indicated that rhizodeposits chemodiversity (%IncMSE = 11.828, *P* = 0.009) than all of soil physicochemical properties (%IncMSEs < 6.474) had greater influence on biodiversity of active bacteria (Fig. S8). The Sørensen’s distances of rhizodeposits were also significantly correlated with that of active bacterial communities (Mantel *r* = 0.570, *P* < 0.001, Fig. 5A), and such a rhizodeposits-microbiome correlation was much higher than the correlation between soil physicochemical properties and active bacterial communities (Mantel *r* = 0.419, *P* < 0.001, Fig. S9). These consistently indicated a tighter association between peanut rhizodeposits and active rhizosphere bacteria.

We then investigated the assemblages of rhizodeposits and active bacteria using phylogeny-informed null model, and the results showed that the assemblages of rhizodeposits were highly governed by deterministic processes, while the active bacteria were mainly controlled by the stochastic processes (Fig. 5B). The correlation between microbial and rhizodeposits betaNTI indices was determined by the Mantel test. Our results showed that betaNTI indices of microbial communities were significantly correlated with the TD-based betaNTI indices of rhizodeposits (Fig. 5C; Mantel *r* = 0.228, *P* = 0.012). This suggested that assembly processes of active bacteria may be significantly associated with the biochemical transformations of rhizodeposits. However, the betaNTI indices between rhizosphere microbiome and rhizodeposits were not significantly correlated when we used the MCD- (Fig. 5D; Mantel *r* = 0.055, *P* = 0.554) or TWCD-based (Fig. 5E; Mantel *r* = 0.026, *P* = 0.779) relational dendrograms, suggesting that molecular properties of rhizodeposits may have a weak influence on the assembly processes of active bacteria. We also tested the potential transformations of rhizodeposits within each sample, and the results indicated that both cultivation duration (*F*_2.18_ = 17.740, *P* = 0.001) and growth stage (*F*_1.18_ = 22.130, *P* = 0.001) had a significant effect on the potential transformations of rhizodeposits, with growth stage playing a bigger role according to the two-way ANOVA analysis (Table S5). Meanwhile, we found a significant decrease in potential transformations of rhizodeposits in P5 than P1 treatment (Fig. 5F), and such a decrease was significantly positively correlated to the contents (Fig. S10; *r* = 0.693, *P* = 0.003) and chemodiversity (Fig. 5G; *r* = 0.983, *P* < 0.001) of rhizodeposits and biodiversity of active bacteria (Fig. 5H; *r* = 0.804, *P* < 0.001).

### Functional potentials of active bacteria

**Fig. 6:**
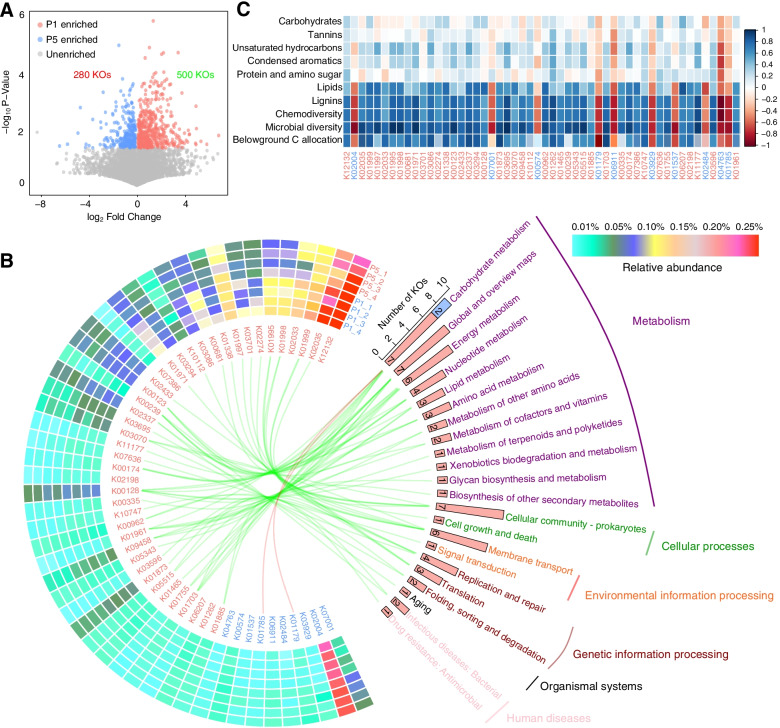
Significantly different KO functional categories between active bacteria of P1 and P5. **A** Depicts all KO functional categories, and the differential KO functional categories were evaluated using the two-tailed Wilcoxon test. **B** Describes the abundant differential KO functional categories (relative abundance > 0.03%), and the involved pathways are counted in the histogram and are linked by lines. **C** Shows the correlations between relative abundance of abundant differential KO functional categories and biodiversity of active bacteria, chemodiversity of rhizodeposits, and relative abundance of main categories of rhizodeposits. P1 and P5 represent samples under continuous monoculture cropping of peanut for 1 and 5 years, respectively

The metagenomic functional profiling yielded a total of 7806 KO functional categories. The PCoA and PERMANOVA indicated that overall microbial functional potential differed significantly between P1 and P5 (*F* = 2.1, *P* = 0.046, Fig. S11). The Mantel test indicated that rhizodeposits composition (Mantel *r* = 0.507, *P* = 0.006) rather than change in soil physicochemical properties (Mantel *r* = 0.094, *P* = 0.635) was the main determinant of the microbial functional potentials (Fig. S12). The microbial functional differences between P1 and P5 were quantified using a two-tailed Wilcoxon test, and a total of 500 and 280 significantly (*P* < 0.05) enriched KO functional categories were discovered in P1 and P5, respectively (Fig. 6A).

To clarify which KO functional categories were most dominant among these differences, the differential KO functional categories with a relative abundance greater than 0.03% are described (Fig. 6B). Fifty-two differential KO functional categories were retained based on this threshold, 42 of which were significantly enriched in P1 while 10 were significantly enriched in P5. In the differential KO functional categories enriched in P1, K12132 (*prkC*, eukaryotic-like serine/threonine-protein kinase [EC: 2.7.11.1]; mean 0.31%) was the most abundant KO followed by K02035 (peptide/nickel transport system substrate-binding protein; mean 0.23%). In P5, K02004 (putative ABC transport system permease protein; mean 0.22%) was the most abundant KO followed by K07001 (NTE family protein; mean 0.07%). We then mapped the abundant differential KOs into KEGG pathways and found that only two KOs that enriched in P5 could be mapped into pathways (carbohydrate metabolism), while all rest KOs that could be mapped into pathways were enriched in P1 (Fig. 6B).

We tested the correlations between abundant differential KOs and soil physicochemical properties, belowground C allocation, species diversity of active bacteria, chemodiversity, and relative abundance of rhizodeposits. We found that most abundant differential KOs were significantly positively correlated with soil pH, soil AP, belowground C allocation, species diversity of active bacteria, chemodiversity of rhizodeposits, and relative abundance of lignins and lipids (Fig. 6C, Fig. S13). However, the correlations between abundant differential KOs and the relative abundance of rest rhizodeposits including protein and amino sugars, condensed aromatics, unsaturated hydrocarbons, tannins, and carbohydrates were relatively weak (Fig. 6C).

**Fig. 7:**
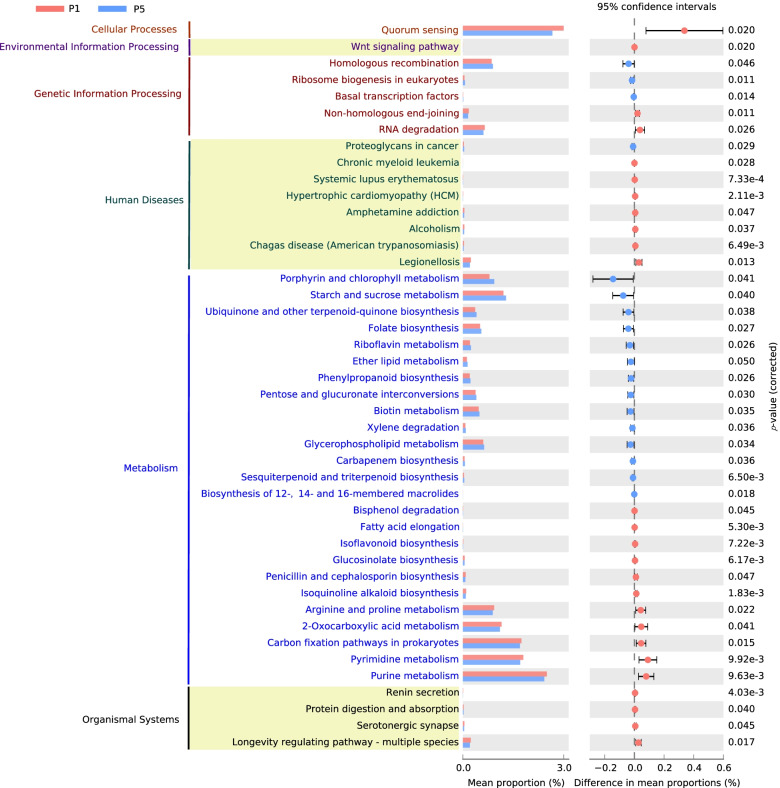
Significantly different KEGG pathways between active bacteria in P1 and P5 treatments. The pathway difference between P1 and P5 was quantified using a two-tailed Wilcoxon test, and the corrected *P*-values were shown. P1 and P5 represent samples under continuous monoculture cropping of peanut for 1 and 5 years, respectively

The KO functional categories were further analyzed for KEGG pathway enrichment, and the results showed that a total of 26 and 18 significantly (*P* < 0.05) enriched KEGG pathways were discovered in P1 and P5, respectively (Fig. 7). The pathways “quorum sensing,” “Wnt signaling pathway,” “nonhomologous end joining,” “RNA degradation,” and almost all differential pathways involved in human diseases were significantly enriched in P1 (*P* < 0.05). The metabolism of arginine and proline, 2-oxocarboxylic acid, pyrimidine, and purine was also enriched in the P1. The biosynthesis pathways of isoflavonoid, glucosinolate, penicillin, cephalosporin, and isoquinoline alkaloid also exhibited significantly increased relative abundances in P1 than P5. Furthermore, some other pathways involved in metabolism such as “bisphenol degradation,” “fatty acid elongation,” and “carbon fixation pathways in prokaryotes” also showed significantly higher relative abundance in the P1. On the contrary, pathways related to genetic information processing such as “homologous recombination,” “ribosome biogenesis in eukaryotes,” and “basal transcription factors” were enriched in P5. The metabolism of porphyrin and chlorophyll, starch and sucrose, riboflavin, ether lipid, biotin, and glycerophospholipid was significantly depleted in P1. Meanwhile, the biosynthesis pathways of ubiquinone and other terpenoid quinone, folate, phenylpropanoid, carbapenem, and sesquiterpenoid and triterpenoid were also significantly depleted in P1. We also tested the correlations between differential KEGG pathways and soil physicochemical properties, biodiversity of active bacteria, chemodiversity of rhizodeposits, and relative abundance of main categories of rhizodeposits and found that more differential KEGG pathways were positively correlated with soil pH, soil AP, species diversity of active bacteria, chemodiversity of rhizodeposits, and relative abundance of lignin and lipids (Fig. S14).

## Discussion

Using state-of-the-art experimental and modeling approaches, the current study demonstrated that the reduced rhizodeposits chemodiversity suppresses the rhizosphere microbiome diversity and functioning in the mono-cropped agroecosystems, which may provide new insight into the role of agricultural intensification in altering the chemodiversity and functioning of microbiome in the soil ecosystems.

Our results clearly indicated that allocation of photosynthesized C was influenced by both plant growth stages and soil management history (mono-cropped duration) (Fig. 2, Table S5), which jointly affected the plant growth. Plant age is the key determinant of the belowground C allocation [41]. The C fixed by photosynthesis is not balanced with the C required for plant growth when plants are small due to higher root-to-shoot ratios [42], thus leading to a higher C input into belowground at W6, whereas older plants preferentially allocate the newly assimilated C to the shoots [20, 43] (Fig. 2B). Besides plant growth, the change in soil nutrients under different continuous cropping durations would also lead to the differential C allocation belowground [18, 41]. Our results indicated that soil nutrients including SOC, TN, AN and AK were positively correlated with belowground C allocation especially at W6 (Table S6), which was consistent with some previous reports that allocation of newly assimilated C to belowground pools was negatively correlated with the amount of mineral nitrogen [17, 41]. Therefore, our results confirmed the resource optimization hypothesis that increasing nutrient availability reduces the C costs of nutrient acquisition [17, 18].

Using FTICR-MS, we investigated the composition and diversity of peanut rhizodeposits. The lignins had highest relative abundance in the peanut rhizodeposits (39–58%), followed by lipids (23–47%) and protein/amino sugars (8–15%) (Fig. 3A). Lignins are important components in the cambium cells of plants and are therefore highly enriched in plant-associated environments such as rhizosphere [44]. We observed significant differences in the composition of rhizodeposits between treatments, and the most important is the chemodiversity of rhizodeposits declined significantly from P1 to P10 treatment (Fig. 3C). We also found a strong positive correlation between plant biomass and the chemodiversity of rhizodeposits (Fig. 3D), thus implying that chemodiversity of rhizodeposits was also highly determined by the plant growth, which was somewhat consistent to some recent findings that root biomass determined the contents and composition of plant rhizodeposits [45, 46].

The DNA-SIP-based amplicon sequencing revealed a significant decrease in biodiversity of active bacteria in peanut rhizosphere from P1 to P5 (Fig. 4), while some recent studies have also predicted that continuous monoculture could alter or suppress microbial diversity in the soil ecosystem [47, 48]. Thus, the declined soil microbial biodiversity in the mono-cropped systems might be an important indicator and driving force beyond the malfunctioning of soil microbiome [3]. Furthermore, our study revealed that many active plant-beneficial microbial species were enriched at P1 such as *Gemmatimonas*, *Streptomyces*, *Ramlibacter*, and *Lysobacter* and at P5 such as *Trinickia* were potentially phytopathogenic, suggesting that the enrichment of plant-harmful microbes was at the expense of plant-beneficial microbes in the mono-cropped system. *Gemmatimonas* play a key role in the release of SOC by degrading complex cellulose and lignin molecules [49, 50] and are important plant-beneficial microbes for multiple leguminous plants such as soybean [51]. *Streptomyces*, *Ramlibacter*, and *Lysobacter* are well-known plant-beneficial bacteria because they can widely control many plant diseases and can promote growth of many plant species including peanut [52, 53]. On the contrary, *Trinickia*, which was significantly enriched in P5, were potentially phytopathogenic [54, 55]. Therefore, the discrepant enrichment of plant-beneficial and phytopathogenic microbes at different cropping duration could be another important indicator and driving force of the microbial-driven continuous cropping obstacle.

The significant correlation between chemodiversity of rhizodeposits and microbial biodiversity (Fig. 5A) suggests the strong impact of rhizodeposits and their composition on active microbial communities in soil. Moreover, the hypothesis *diversity begets diversity* predicts that higher resource diversity can provide wider habitat niche breadth for organisms and can therefore promote higher biodiversity [30]. If this is true, the resource diversity should be positively correlated with the biodiversity. Such a correlation has been predicted only in few naturel ecosystems such as rivers and lakes [30, 32, 56]. Therefore, our results not only confirmed the *diversity begets diversity* hypothesis but also extended this idea to the dynamic plant-microbe system. Using phylogeny-informed models, we instigated the ecological processes underlying the assembly of rhizodeposits and active bacterial communities (Fig. 5B). We initially expected that the assembly processes of rhizodeposits and active bacterial community would be consistent due to their intimate association [57], and the processes driving rhizodeposits would thereafter be the same for microbial community as well. Surprisingly, we found that the assembly processes of rhizodeposits and active bacterial communities were highly contrasting, with rhizodeposits being absolutely governed by deterministic processes (variable selection) while active bacterial communities being mainly controlled by the stochastic processes. This implies that continuous monoculture may have much strong effects on the rhizodeposits than microbial communities, possibly because the variation in microbial communities during mono-cropping is also determined by some stochastic processes (e.g., stochastic dispersal, diversification, and ecological drift) besides the selection of rhizodeposits. Although rhizodeposits and microbial communities were governed by contrasting assembly processes, we also observed a significant positive correlation between the potential transformations of rhizodeposits and the active microbial community assembly processes, thus suggesting that the assembly processes acting upon rhizodeposits would also reflect on microbial community assemblages. Specifically, if the rhizodeposits transform due to more deterministic processes, the associated active microbial community also shifts more deterministically (Fig. 5C).

Moreover, the DNA-SIP-based metagenome sequencing showed that more KOs were significantly enriched in P1, thus implying a decrease in the functional potential of active bacteria especially those involved in metabolism during continuous cropping (Fig. 6). The change in the functional abundance of active bacteria was highly correlated with belowground C allocation, microbial biodiversity, and chemodiversity of the rhizodeposits (Fig. 6C). Although microbes are highly functionally redundant [58, 59], the massive loss of microbial species, especially rare microbial species, which play a key role in maintaining the microbial functional pool [60], may also result in the functional loss [61, 62]. Consequently, the significant decrease in the microbial biodiversity may suppress microbiome functional potential during continuous cropping. Given that rhizodeposits are substrates for many rhizosphere microorganisms to function [25], the microbial functional potentials were suppressed when the chemodiversity of rhizodeposits was declined (Fig. 6). We also found that microbial functional potentials were strongly correlated to abundant rhizodeposits belonging to lignins and lipids, thus suggesting that functional potentials of rhizosphere microbial communities were mainly mediated by abundant rather than rare rhizodeposits (Fig. 6C).

Some KOs and pathways that were differentially enriched in P1 or P5 were important to soil health and plant growth. For example, “quorum sensing” (QS) pathway, enriched in P1, is the key pathway of microbe-microbe communication [63, 64], and these QS signal-deficient microbes can hardly colonize the host plants [65–67]. The QS also has plant-growth-promotion effects, which has been observed on rice [68] and *Arabidopsis* [69]. The K02035, a member of the quorum sensing pathway, was also significantly enriched in P1. Moreover, the pathways involved in the biosynthesis of penicillin, cephalosporin, and isoquinoline alkaloid were also significantly enriched in the P1 (Fig. 7). Penicillin, cephalosporin, and isoquinoline alkaloid are well known and commonly used antibiotics, which can suppress soil-borne pathogens to a great extent [70, 71]. This will prevent the invasion of pathogens, thus keeping plant healthy. Some pathways enriched in P5 such as the biosynthesis of sesquiterpenoid and triterpenoid were also associated with soil and plant health. A previous study found that 52% of *Arabidopsis*-specific root microbiomes were mediated by triterpenoid, thus suggesting the very strong selection of triterpenoid for the soil microbiome [72], which may also explain the decreased biodiversity under continuous mono-cropping.

## Conclusions

Using ^13^CO_2_ labeling, metabolomic and metagenomic analyses, we demonstrated that the significant decrease in the chemodiversity of rhizodeposits, active rhizosphere microbial biodiversity, and functional pathways or traits was key indicators of reduced microbiome functioning in the rhizosphere ecosystems. More importantly, we demonstrated that the reduction in C deposition and chemodiversity in rhizosphere tended to suppress microbial biodiversity and its functions. Our findings provide novel insights into the role of rhizosphere chemodiversity in influencing the rhizosphere microbial biodiversity and its functions, which are essential for soil ecosystem functioning.

## Supplementary Information

**Additional file 1: **Supplementary information. **Text S1**: Calculation of the C allocation. **Text S2**: FTICR-MS sample preparation. **Text S3**: Isopycnic density gradient centrifugation and quantitative PCR. **Text S4**: Amplicon high-throughput sequencing data processing. **Text S5**: Metagenome sequencing and data processing. **Text S6**: Detailed information about data analyses. **Figure S1**: Assessment of labeling, centrifugation, and sequencing. **Figure S2**: The relational dendrograms of rhizodeposits. **Figure S3**: Relative abundance of peanut rhizodeposits. **Figure S4**: Correlations between the relative abundance of main categories of peanut rhizodeposits. **Figure S5**: Species composition of active bacterial communities. **Figure S6**: Correlation between belowground carbon allocation and species richness of active bacteria. **Figure S7**: Significantly different bacterial genera between P1 and P5 treatments. **Figure S8**: Random Forest model determining the key factors affecting the biodiversity of active rhizosphere microbiome. **Figure S9**: Mantel test investigating the relationship between microbial structure and changes in soil physicochemical properties. **Figure S10**: Correlation between potential transformations and concentration of peanut rhizodeposits. **Figure S11**: Principal coordinate analysis on the functional potentials of active bacterial communities. **Figure S12**: Mantel test investigating the relationship between functional potentials of active bacterial communities and rhizodeposits composition and changes in soil physicochemical properties. **Figure S13**: Correlations between relative abundance of abundant differential KO functional categories and soil physicochemical properties. **Figure S14**: Correlation between relative abundance of differential KEGG pathways and biodiversity of active bacteria, chemodiversity of rhizodeposits, and relative abundance of main categories of rhizodeposits. **Table S1**: Physicochemical properties of experimental soils. **Table S2**: The PERMANOVA investigating the structural difference of bacterial community in different CsCl buoyant density gradient of DNA fractions. **Table S3**: Detailed information about primers and PCR conditions. **Table S4**: Data size of metagenome sequences. **Table S5**: Two-way ANOVA investigating the factors affecting the belowground photosynthesized C allocation, chemodiversity and potential transformations of peanut rhizodeposits. **Table S6**: Correlations between belowground C allocation and plant and soil physicochemical properties. **Table S7**: Correlations between soil physicochemical properties and rhizodeposits chemodiversity. **Table S8**: Two-way PERMANOVA investigating the factors affecting the composition of rhizodeposits and structure of the active bacterial community in the peanut rhizosphere.

## Data Availability

The raw DNA-SIP-based 16S rRNA gene sequences and metagenomic sequencing data are available in the NCBI Sequence Read Archive (SRA) under the accession numbers SRP348039 and SRP347853, respectively.
