# Supplementary material for: Reduced chemodiversity suppresses rhizosphere microbiome functioning in the mono-cropped agroecosystems
Source: Microbiome. 2022 Jul 16;10:108. doi: 10.1186/s40168-022-01287-y (PMC9287909; doi:10.1186/s40168-022-01287-y)
Supplement: Supplementary file 2 — Additional file 1: Supplementary information. Text S1: Calculation of the C allocation. Text S2: FTICR-MS sample preparation. Text S3: Isopycnic density gradient centrifugation and quantitative PCR. Text S4: Amplicon high-throughput sequencing data processing. Text S5: Metagenome sequencing and data processing. Text S6: Detailed information about data analyses. Figure S1: Assessment of labeling, centrifugation, and sequencing. Figure S2: The relational dendrograms of rhizodeposits. Figure S3: Relative abundance of peanut rhizodeposits. Figure S4: Correlations between the relative abundance of main categories of peanut rhizodeposits. Figure S5: Species composition of active bacterial communities. Figure S6: Correlation between belowground carbon allocation and species richness of active bacteria. Figure S7: Significantly different bacterial genera between P1 and P5 treatments. Figure S8: Random Forest model determining the key factors affecting the biodiversity of active rhizosphere microbiome. Figure S9: Mantel test investigating the relationship between microbial structure and changes in soil physicochemical properties. Figure S10: Correlation between potential transformations and concentration of peanut rhizodeposits. Figure S11: Principal coordinate analysis on the functional potentials of active bacterial communities. Figure S12: Mantel test investigating the relationship between functional potentials of active bacterial communities and rhizodeposits composition and changes in soil physicochemical properties. Figure S13: Correlations between relative abundance of abundant differential KO functional categories and soil physicochemical properties. Figure S14: Correlation between relative abundance of differential KEGG pathways and biodiversity of active bacteria, chemodiversity of rhizodeposits, and relative abundance of main categories of rhizodeposits. Table S1: Physicochemical properties of experimental soils. Table S2: The PERMANOVA investigating the structura [file 40168_2022_1287_MOESM2_ESM.pdf]

Supplementary information for

Reduced chemodiversity suppresses rhizosphere microbiome functioning in the mono-cropped agroecosystems

Pengfa Li[1,2], Jia Liu[3], Muhammad Saleem[4], Guilong Li[1], Lu Luan[1], Meng Wu[1], Zhongpei Li[1]

[1] State Key Laboratory of Soil and Sustainable Agriculture, Institute of Soil Science, Chinese Academy of Sciences, Nanjing 210008, China

[2] Department of Microbiology, Key Lab of Microbiology for Agricultural Environment, Ministry of Agriculture, College of Life Sciences, Nanjing Agricultural University, Nanjing 210095, China

[3] Soil and Fertilizer & Resources and Environment Institute, Jiangxi Academy of Agricultural Sciences, Nanchang 330200, China

[4] Department of Biological Sciences, Alabama State University, Montgomery, AL 36104, USA

* **Corresponding author**:

Meng Wu, mwu@issas.ac.cn;

Zhongpei Li, zhpli@issas.ac.cn.

This PDF file includes:

Supplementary Text S1 to S6;

Supplementary Figure S1 to S14;

Supplementary Table S1 to S8.

Supplementary materials and methods

Text S1. Calculation of the C allocation

The amount of photosynthesized ^{13}C accumulated in the plant organs (shoots and roots) and soils were calculated using Equation 1 [1]:

$$^{13}C_{amount(a)}=[(atom^{13}C\%)_{l}-(atom^{13}C\%)_{nl}]_{a}\times TC_{a}/100 \quad (1)$$

where "l" and "nl" are labeled and non-labeled samples, respectively; "a" refers to a plant organ (shoots and roots) or soil fraction; and "TC_a" is the total carbon content (mg kg^{-1}) of a.

Net assimilated ^{13}C was calculated using Equation 2:

$$Net13C_{assimilation}(mg^{13}C)={}^{13}C_{shoots}+{}^{13}C_{root}+{}^{13}C_{soil} \quad (2)$$

The photosynthesized ^{13}C distribution in plant and soil was calculated as follows:

$$^{13}C_{proportion}(a)={}^{13}C_{amount}(a)/Net^{13}C_{assimilation}\times 100 \quad (3)$$

Text S2. FTICR-MS sample preparation

The rhizodeposit solution were filtered through 0.45-μm membrane filter. Then the rhizodeposits were extracted from the solution using ethyl acetate on a vacuum rotary evaporator and dissolved in 1.5 mL methanol. The HPLC grade methanol (10 mL) and acidified ultrapure water (10 mL, pH = 2) were passed through the PPL cartridges (Agilent Technologies, Santa Clara, CA, USA) before analysis to clean the cartridges. Then, the rhizodeposit samples were loaded onto PPL cartridges. After that, the rhizodeposits were collected from the PPL cartridges using 10 mL of methanol (HPLC grade; Merck, Germany). The elutes of rhizodeposits were kept at -20°C in the dark prior to the electrospray ionization Fourier transform ion cyclotron resonance mass spectrometry (ESI FTICR-MS) measurements.

We added deuterated octadecanoic acid as an internal standard with a dose of 15 μL (5×10^{-7} mol/L) per milliliter of the rhizodeposits samples. The ESI FTICR-MS (Bruker, Billerica, MA, USA) was equipped with a 9.4 T superconducting magnet interfaced with the negative-ion mode electrospray ionization. We injected each sample into the ESI source at the speed of 180 μL/h using a syringe pump. The polarization voltage was the 4.0 kV. The capillary column, introduction, and outlet, voltages were 4.5 kV and 320V, respectively. The ions accumulated in the hexapole for 0.001s before were transferred to the ICR cell. The m/z range was 150–800 Da. A 4M word size was selected for the time

domain signal acquisition. The signal to noise ratio and dynamic range were enhanced through accumulating 128 times domain FTICR transients.

Text S3. Isopycnic density gradient centrifugation and quantitative PCR

Total soil DNA was extracted from 0.5 g soil using a FastDNA SPIN Kit for Soil (MP Biomedicals, Cleveland, OH, USA) according to the manufacturer's instructions. The purity and quantity of DNA were assessed using a Nanodrop One^{C} UV-Vis Spectrophotometer (Thermo Fisher Scientific, Waltham, MA, USA). Briefly, 3.0 µg of DNA extract was mixed with CsCl solution with an initial buoyant density adjusted to 1.725 g ml^{-1} using the gradient buffer (100 mM tris HCl, 100 mM KCl, 1.0 mM EDTA, pH 8.0). The mixture was then added to a 5.1-ml Beckman polyallomer ultracentrifuge tube before centrifugation at 20 °C in a VTi65.2 vertical rotor (Beckman Coulter, Palo Alto, CA, USA) at 177,000 x g for 44 h. The gradient solution in each tube was fractionated from bottom to top by displacement with sterile water using an NE-1000 single syringe pump (New Era Pump Systems Inc., Farmingdale, NY, USA). Fifteen DNA fractions (approximately 340 µl each) were obtained for each sample, and the buoyant density of each fraction was determined by the refractive index using an AR200 digital hand-held refractometer (Reichert, Buffalo, NY, USA). The fractionated DNA was then precipitated using PEG 6000 for 2 h followed by centrifugation at 13,000 x g for 30 min. The DNA pellet was washed twice with 70% ethanol and finally dissolved in 30 µl TE buffer.

The 16S rRNA gene abundances from the DNA of each fraction isolated by the gradient centrifugation were quantified by quantitative PCR on a CFX96 Optical

Real-Time Detection System (Bio-Rad, Hercules, CA, USA). The reaction was performed in a 20-μl mixture containing 10 μl SYBR Premix Ex Taq (Takara Biotech, Dalian, Liaoning, China), 0.5 μM of each primer, and 1 μl of DNA template. The primer pair 515F/907R was used to quantify the 16S rRNA gene, and the quantitative PCR conditions were as follows: 95 °C for 3 min, followed by 39 cycles of 95 °C for 30 s, 55 °C for 30 s, and 72 °C for 30 s [2]. Melting curve analysis was performed for every PCR analysis to confirm the specificity of the amplification products by continuously measuring the fluorescence as the temperature increased from 65 to 95 °C.

The relative abundance of 16S rRNA gene in the rhizosphere soils of unlabeled peanut plants (^{12}C-DNA) peaked at the buoyant density of 1.70 g mL^{-1} (light fraction) at both growth stages (W6 & W10); while that of labeled peanut plants (^{13}C-DNA) peaked at the buoyant density of 1.71 g mL^{-1} and 1.72 g mL^{-1} (heavy fraction) at W6 and W10, respectively (Fig. S1). The PCoA combined with the PERMANOVA based on the Bray-Curtis dissimilarity of bacterial communities indicated significant differences between the DNA groups (Fig. S1, Table S2). These results clearly indicated that the 16S rRNA genes were successfully labeled, enriched, and separated by the isopycnic ultracentrifugation. The uncentrifuged DNA (raw DNA) had highest species richness, followed by ^{13}C light fractions, while ^{13}C light fractions, ^{12}C heavy fractions and ^{12}C light fractions showed no significant differences in the species richness (Fig. S1).

Text S4. Amplicon high-throughput sequencing data processing

The raw sequence data were analyzed using the QIIME 2(version 2021.8) [3]. Raw sequence data were demultiplexed and quality filtered using the q2-demux plugin followed by denoising with DADA2 (via q2-dada2) [4], and the sequences that were not present in at least 2 samples were filtered out. After quality filtering and the removal of chimaeras, sequences were clustered into 2991 ASVs after rarefying to 15,333 sequences per sample (based on the sample with the minimum numbers of reads) [5]. The taxonomic assignment of representative sequences was performed using RDP classifier (http://rdp.cme.msu.edu/classifier/) with 80% confidence threshold [6]. A phylogenetic tree was first constructed using the Fasttree [7] after aligning the representative OTU sequences using PyNAST [8].

Text S5. Metagenome sequencing and data processing

The density-gradient centrifugation was conducted on DNA sample from each rhizosphere soil sample, and ^{13}C-DNA from the "heavy" gradient fractions was pooled to obtain sufficient ^{13}C-DNA for the metagenomics sequencing. Paired-end reads (2 × 150 bp) were generated on the Illumina Hiseq 4000 platform (Illumina Inc., San Diego, CA, USA). A total of 70.0–86.7 million raw reads per sample were yielded, with each sampling containing about 11.83 Gb raw data. The resulting sequence reads were analyzed for quality control using a range of software programs: SeqPrep (https://github.com/jstjohn/SeqPrep) for tripping nonbiological bases in reads, such as primers or barcodes, and Sickle (https://github.com/najoshi/sickle) for filtering reads whose length after tripping was less than 50 bp and whose average quality score was less than 20. Then, 68.3–83.5 million clean reads per sample were obtained. The optimized sequence reads were assembled de novo by SOAPdenovo (http://soap.genomics.org.cn/, Version 1.06) based on a de Bruijn graph for obtaining contigs, and a total of 6,370,273 contigs were generated. Resulting contigs > 500 bp in length were selected to predict open reading frames (ORFs) using MetaGene (http://metagene.cb.k.u-tokyo.ac.jp/), and 8,374,946 ORFs were obtained. All predicted genes were aligned pairwise using CD-HIT (http://www.bioinformatics.org/cd-hit/) [9], and those for which more than 90% of their length could be aligned to another gene with more than 95% identity (no gaps allowed) were removed to generate the non-redundant gene catalog.

The high-quality reads from each sample were aligned against the gene catalog by SOAPaligner (http://soap.genomics.org.cn/) using the criterion "identity > 95%." In each sample, the mapped reads of each gene were counted as the number of gene-mapped reads, and the gene relative abundance was calculated following a previous study. We aligned putative amino acid sequences, which were translated from the gene catalog, against the proteins/domains in KEGG databases (Release 79.0) using BLASTP (BLAST v. 2.2.28+, http://blast.ncbi.nlm.nih.gov/Blast.cgi) (e value ≤ 1e-5). The current study yielded 7806 KOs and 409 KEGG pathways. The abundance of any KO was calculated following the equation below [10].

$$a_i = \frac{b_i}{\sum_j b_j} = \frac{\frac{x_i}{L_i}}{\sum_i \frac{x_j}{L_j}}$$

Where ai is the relative abundance of gene *i* in sample S; L_i represents the length of gene *i*; x_i is the times at which gene *i* could be detected in sample S (the number of mapped reads); and b_i is the copy number of gene *i* in the sequenced data from sample S.

Text S6. Detailed information about data analyses

The R package '*car*' was used to analyze the normality of data distribution (Shapiro-Wilk test) and homogeneity of the variance (Levene's test). Then, the significant differences between treatments were determined using one-way analysis of variance (ANOVA), followed by Duncan's test for multiple comparisons ($P < 0.05$). However, if data were not normally distributed and/or variances were heterogeneous, the two-tailed Wilcoxon rank-sum test was performed to determine the statistical differences between treatments.

Three relational dendrograms based on molecular characteristics (MCD), potential biochemical transformations (TD), and transformation-weighted characteristics (TWCD) of the rhizodeposits were constructed [11]. Briefly, we calculated the Euclidean distances among rhizodeposits based on the elemental composition (including C, H, O, N, O:C ratio, H:C ratio and N:C ratio), double-bond equivalents, modified aromaticity index, and Kendrick's defect to construct the MCD using the UPGMA method. The TD measures molecular similarity by estimating the potential biochemical transformations on the basis of ultrahigh mass resolution variations between the identified rhizodeposits. We constructed a transformation network between rhizodeposits using the pairwise mass differences, while the correlations between rhizodeposits were calculated by picking the largest cluster of interconnected nodes (every node denotes an individual rhizodeposits molecule). Then, we measured the stepwise distance

between each pair of the soil rhizodeposits. Afterwards, the TD was constructed using the UPGMA method, based on the standardized Euclidean distances. After combing the molecular characteristics and standardized transformation matrices, we constructed TWCD using the UPGMA method.

After getting the relational dendrograms of rhizodeposits, we used an ecological assembly model (the so-called betaNTI, dendrogram-based) to investigate the assembly processes governing the composition of rhizodeposits and active microbial communities [12]. Briefly, the betaNTI was calculated as follows:

$$\mathrm{betaNTI} = -1\left(\frac{\mathrm{betaMNTD_{obs}} - \overline{\mathrm{betaMNTD_{null}}}}{\mathrm{SD(betaMNTD_{null})}}\right)$$

where the betaMNTDobs is the observed betaMNTD for the observed assemblages, while the $\overline{\mathrm{betaMNTD_{null}}}$ is the average betaMNTD for the null communities. The SD($\mathrm{betaMNTD_{null}}$) is the standard deviation of the $\mathrm{betaMNTD_{null}}$ values.

Supplementary figures

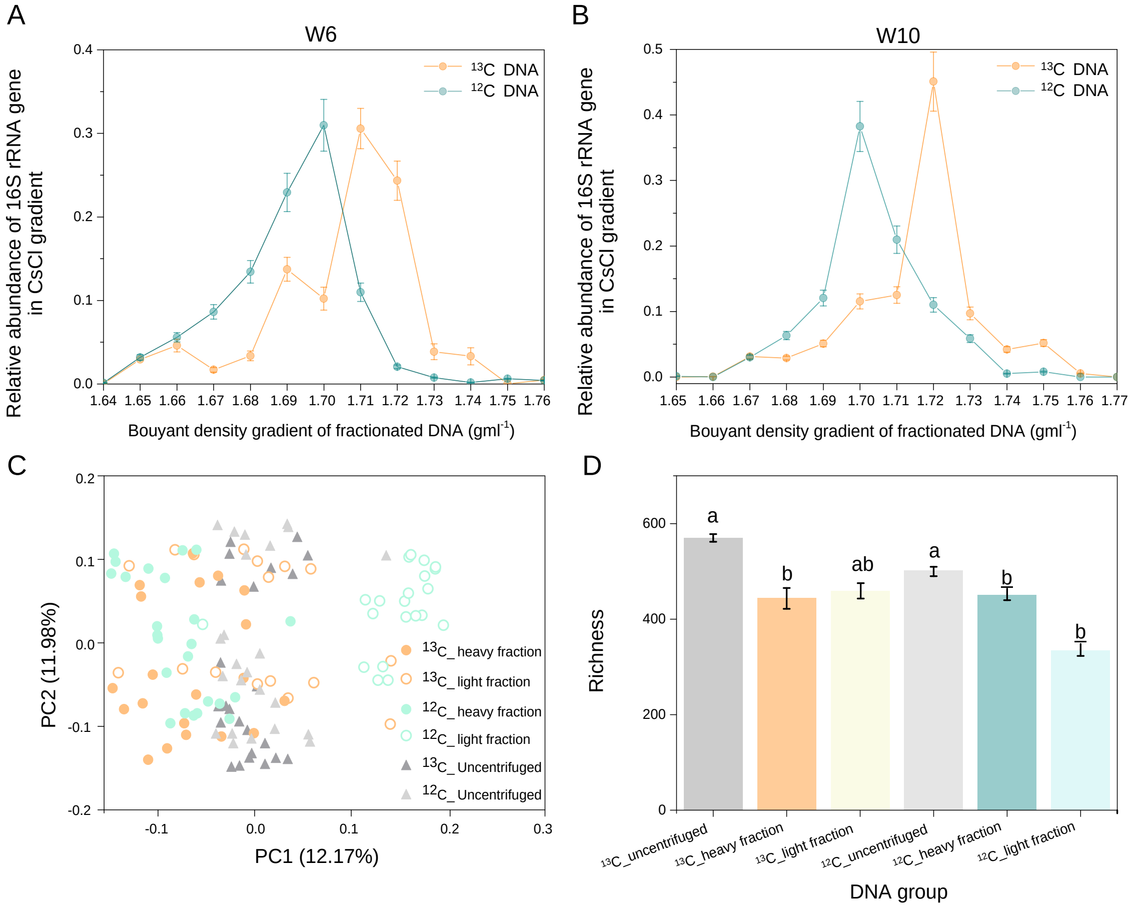

Figure S1. Assessment of labeling, centrifugation, and sequencing. (A-B) Quantitative distribution of density-resolved 16S rRNA gene across entire buoyant density gradient of DNA fractions from rhizosphere soils of unlabeled and labeled peanut plants at W6 and W10. (C) The principal coordinates analysis on the composition of bacterial communities in different DNA groups (based on Bray-Curtis dissimilarity). (D) The species richness of bacterial communities in different DNA groups. Different lowercases indicate significant difference among groups at P <0.05.

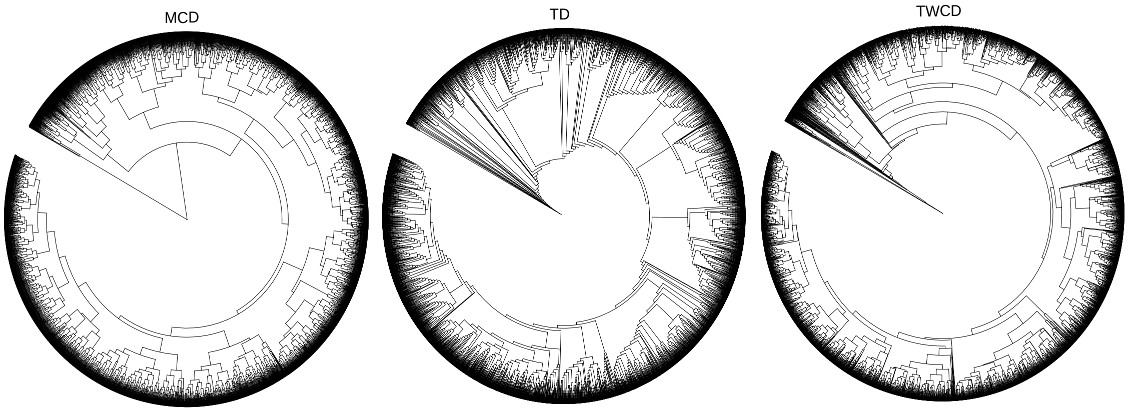

Figure S2. The relational dendrograms of rhizodeposits. MCD, TD and TWCD represents the relational dendrogram constructed based on molecular characteristics (MCD), potential biochemical transformations (TD), and transformation-weighted characteristics (TWCD) of the rhizodeposits, respectively.

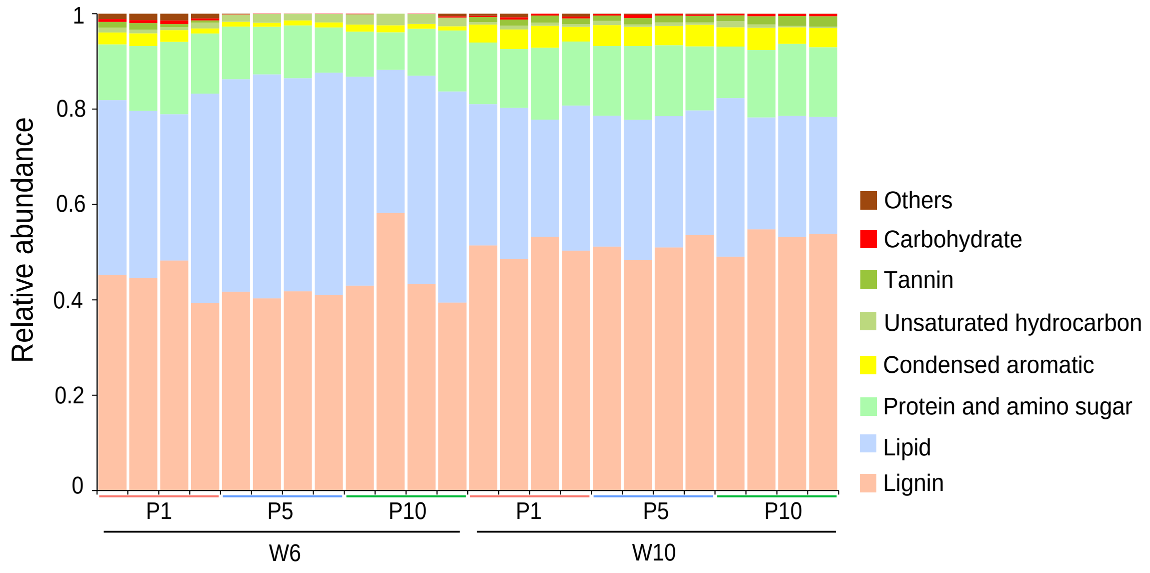

Figure S3. Relative abundance of peanut rhizodeposits.

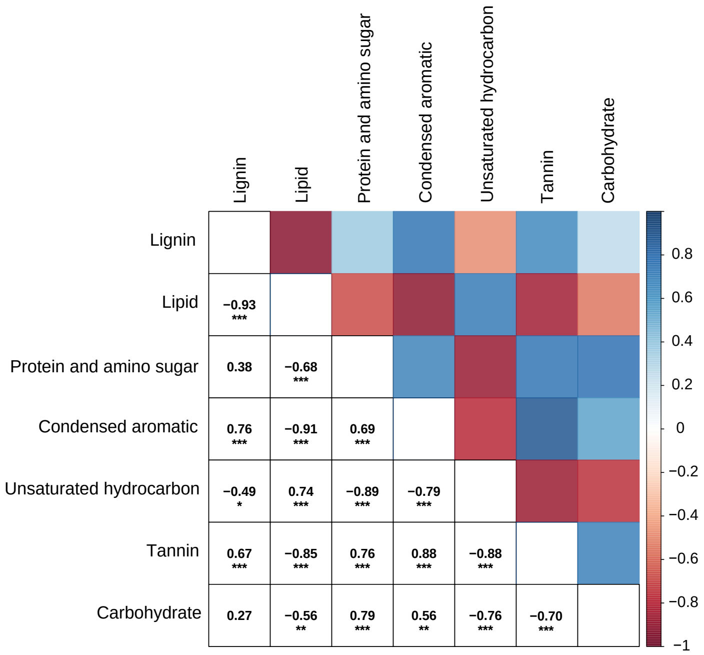

Figure S4. Correlations between the relative abundance of main categories of peanut rhizodeposits. *, **, and *** indicates a significant correlation at $P <$ 0.05, 0.01, and 0.001, respectively.

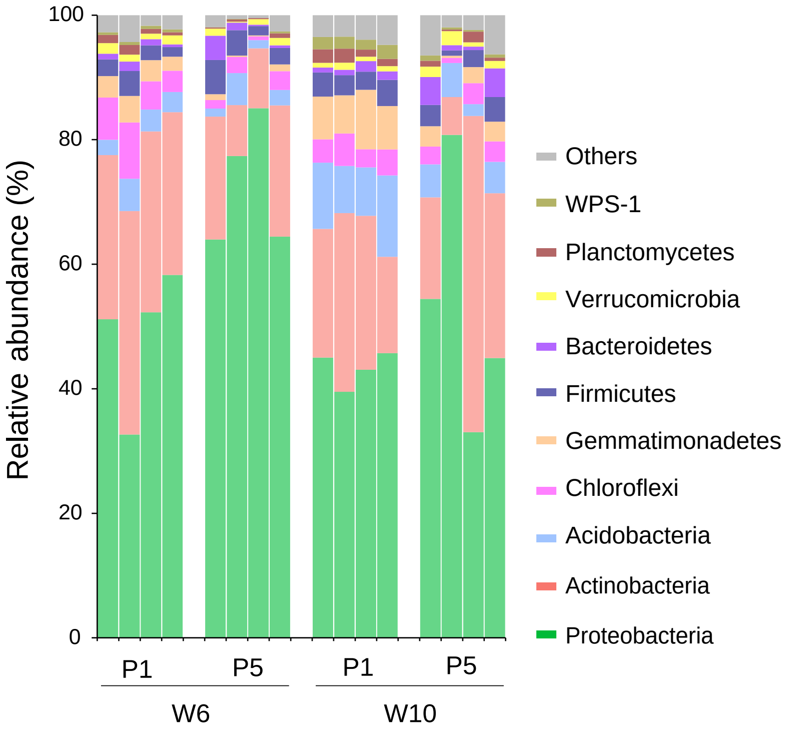

Figure S5. Species composition of active bacterial communities. Based on the average relative abundance, the top ten bacterial phyla were shown.

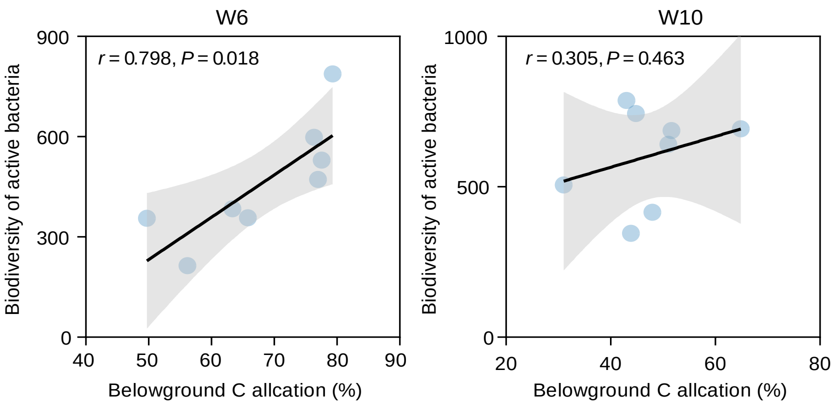

Figure S6. Correlation between belowground carbon allocation and species richness of active bacteria. Lines represent the least squares regression fits and shaded areas represent the 95% confidence intervals. We applied one-side *F* and two-side *t* tests, and then calculated *P* values as shown.

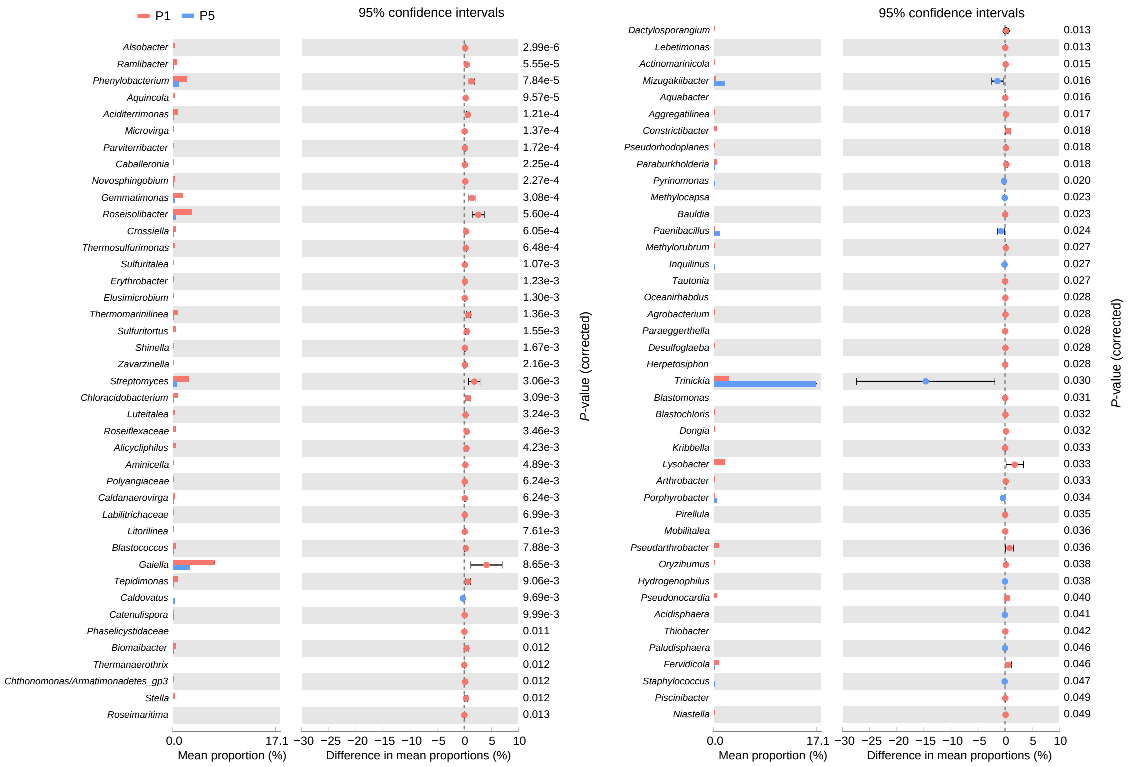

Figure S7. Significantly different bacterial genera between P1 and P5 treatments. The genus difference between P1 and P5 were quantified using a two-tailed Wilcoxon test based on relative abundance, and the corrected *P* values were shown. P1 and P5 represent samples under continuous monoculture cropping of peanut for 1 and 5 years, respectively.

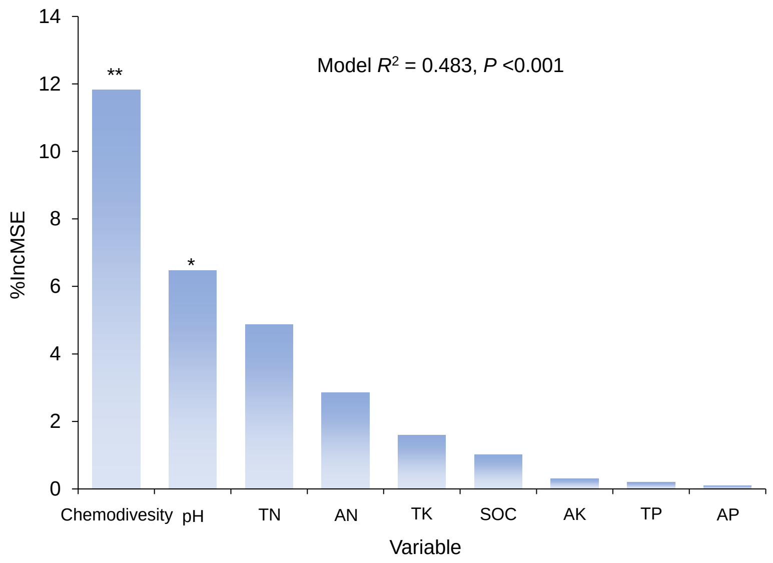

Figure S8. Random Forest model determining the key factors affecting the biodiversity of active rhizosphere microbiome. The importance of each predictor was determined by assessing the decrease in prediction accuracy [that is, the increase in the mean square error (%IncMSE) between observations and predictions] when the data for the predictor was randomly permuted. This decrease was averaged over all trees to produce the final measure of importance. ∗, ∗∗, and ∗∗∗ represent significant %IncMSE at P < 0.05, 0.01, and 0.001, respectively. SOC: soil organic carbon; TN: total nitrogen; TP: total phosphorus; TK: total potassium; AN: available nitrogen; AP: available phosphorus; AK: available potassium.

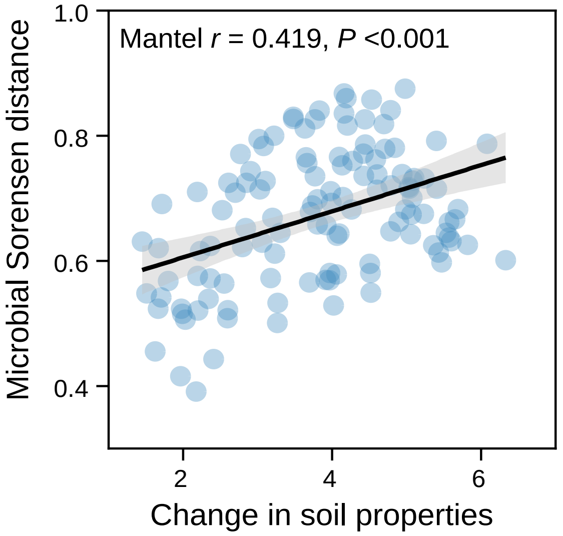

Figure S9. Mantel test investigating the relationship between microbial structure and changes in soil physicochemical properties. The changes in soil physicochemical properties were calculated based on the variance-covariance matrix of all soil physicochemical properties. The data of soil physicochemical properties were standardized to a mean of 0 and SD of 1.

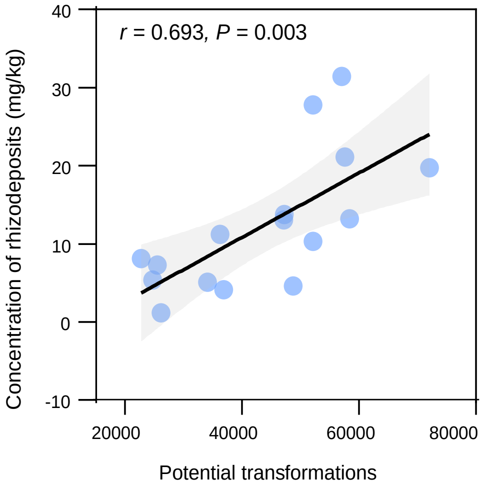

Figure S10. Correlation between potential transformations and concentration of peanut rhizodeposits. Lines represent the least squares regression fits and shaded areas represent the 95% confidence intervals. We applied one-side *F* and two-side *t* tests, and then calculated *P* values as shown.

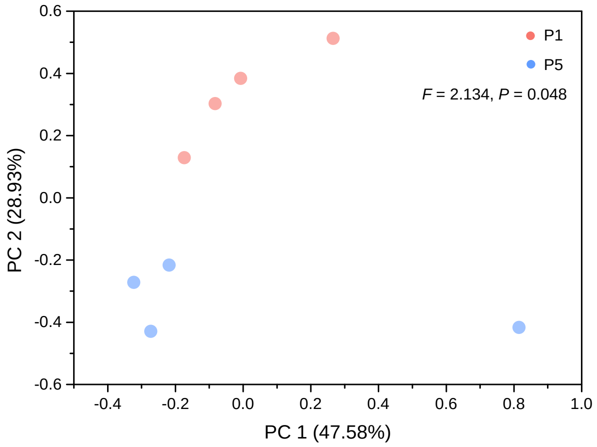

Figure S11. Principal coordinate analysis on the functional potentials of active bacterial communities. The PERMANOVA was conducted to investigate whether the functional potentials of active bacterial communities were significantly different between treatments.

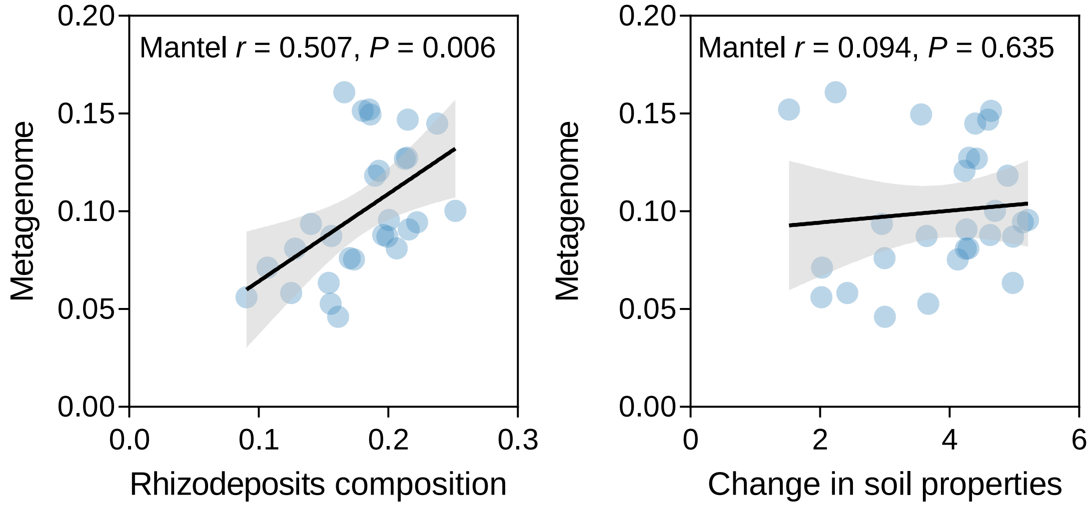

Figure S12. Mantel test investigating the relationship between functional potentials of active bacterial communities and rhizodeposits composition and changes in soil physicochemical properties. The changes in soil physicochemical properties were calculated based on the variance-covariance matrix of all soil physicochemical properties. The data of soil physicochemical properties were standardized to a mean of 0 and SD of 1.

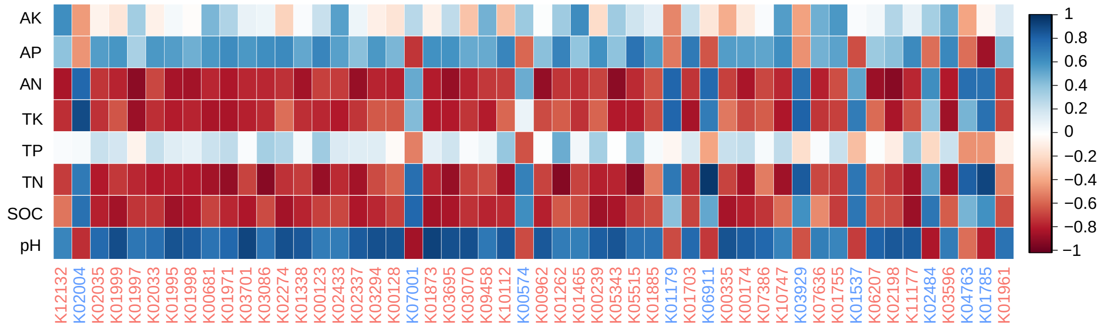

Figure S13. Correlations between relative abundance of abundant differential KO functional categories and soil physicochemical properties. SOC: soil organic carbon; TN: total nitrogen; TP: total phosphorus; TK: total potassium; AN: available nitrogen; AP: available phosphorus; AK: available potassium.

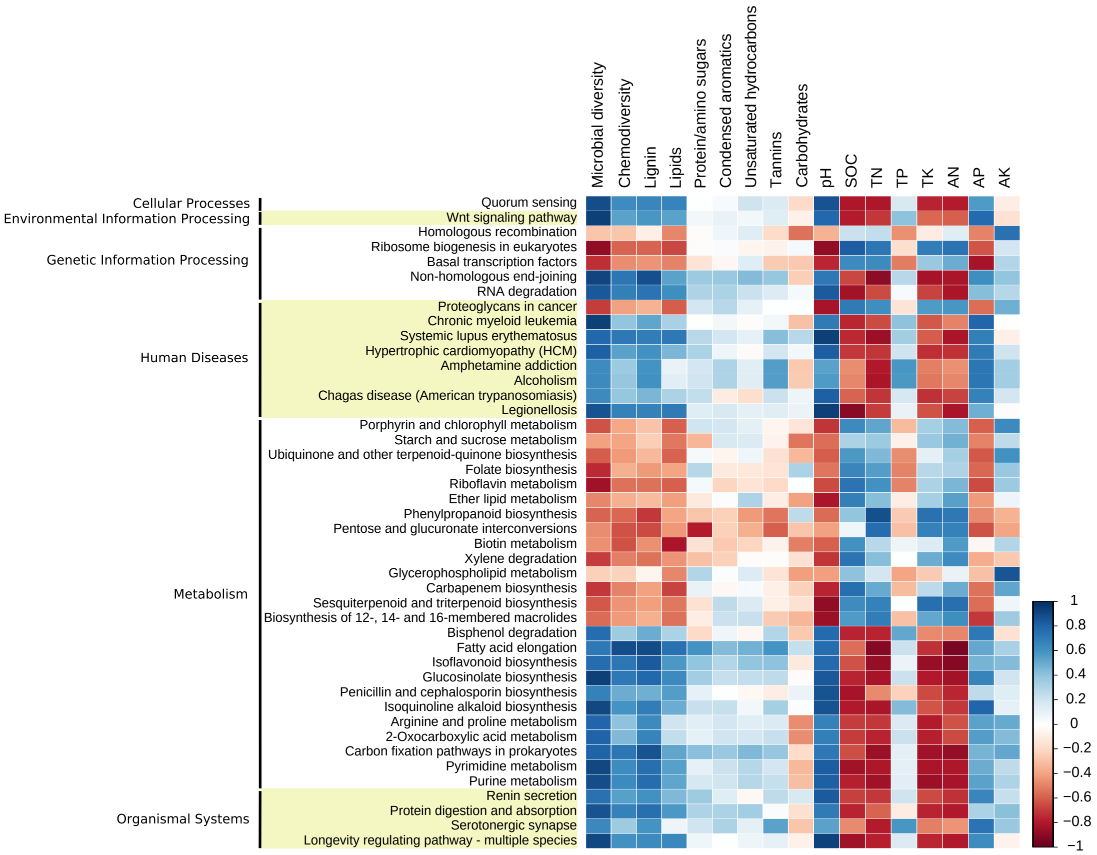

Figure S14. Correlation between relative abundance of differential KEGG pathways and soil physicochemical properties, biodiversity of active bacteria, chemodiversity of rhizodeposits, and relative abundance of main categories of rhizodeposits. SOC: soil organic carbon; TN: total nitrogen; TP: total phosphorus; TK: total potassium; AN: available nitrogen; AP: available phosphorus; AK: available potassium.

Supplementary tables

Table S1. Physicochemical properties of experimental soils. The upper panel shows the physicochemical properties of the field soil; the middle and bottom panels show the physicochemical properties of rhizosphere soil in pot experiment at W6 and W10, respectively. Data in the middle and bottom panels are mean ± standard error. Different lowercases indicate significant difference under $P < 0.05$. SOC: soil organic carbon; TN: total nitrogen; TP: total phosphorus; TK: total potassium; AN: available nitrogen; AP: available phosphorus; AK: available potassium. P1, P5, and P10 represent the soil that has been continuously cultivated for one, five, and ten years, respectively.

	P1	P5	P10
Field soil			
pH	5.20	4.71	4.41
SOC (g/kg)	8.70	11.78	11.31
TN (g/kg)	0.92	1.24	1.25
TP (g/kg)	0.68	0.72	0.65
TK (g/kg)	11.51	14.41	12.92
AN (mg/kg)	99.01	120.11	162.02
AP (mg/kg)	37.81	29.92	11.70
AK (mg/kg)	104.13	194.05	108.30

Rhizosphere soil at W6			
pH	5.72±0.03 a	5.22±0.05 b	4.97±0.10 b
SOC (g/kg)	9.61±0.29 b	10.97±0.19 ab	11.95±0.22 a
TN (g/kg)	1.01±0.04 b	1.25±0.03 a	1.23±0.02 a
TP (g/kg)	0.61±0.00 a	0.63±0.01 a	0.63±0.01 a
TK (g/kg)	11.19±0.28 a	12.20±0.54 a	11.79±0.51 a
AN (mg/kg)	101.82±2.53 b	117.25±5.30 ab	152.13±6.56 a
AP (mg/kg)	42.25±1.36 a	37.05±2.33 ab	33.27±2.48 b
AK (mg/kg)	334.49±6.10 a	374.67±23.11 a	361.50±7.79 a
Rhizosphere soil at W10			
pH	5.61±0.06 a	5.16±0.05 b	4.94±0.11 b
SOC (g/kg)	10.45±0.24 b	11.70±0.29 ab	12.71±0.16 a
TN (g/kg)	1.04±0.03 b	1.25±0.04 a	1.27±0.03 a
TP (g/kg)	0.63±0.01 a	0.61±0.01 a	0.62±0.02 a
TK (g/kg)	11.72±0.28 a	12.91±0.37 a	12.57±0.33 a
AN (mg/kg)	104.78±2.87 b	121.63±3.90 ab	149.25±6.55 a
AP (mg/kg)	37.80±1.01 a	33.48±1.47 ab	31.99±1.89 b
AK (mg/kg)	368.60±28.26 a	356.54±34.73 a	388.04±23.73 a

Table S2. The PERMANOVA investigating the structural difference of bacterial community in different CsCl buoyant density gradient of DNA fractions. The lower and upper triangular matrix shows the *F* and *P* values of PERMANOVA test, respectively.

	^{13}C_heavy	^{13}C_light	^{12}C_heavy	^{12}C_light
^{13}C_heavy		<0.001	<0.001	<0.001
^{13}C_light	8.382		<0.001	<0.001
^{12}C_heavy	7.577	15.760		<0.001
^{12}C_light	19.710	10.920	28.150	

Table S3. Detailed information about primers and PCR conditions.

Primers	515F (GTGCCAGCMGCCGCGGTAA)<br><br>907R (CCGTCAATTCCTTTGAGTTT)
Target gene	16S
Target subfragment	V4-V5
PCR reaction condition	An initial denaturation at 95 °C for 3 minutes, followed by 27 cycles of 30 s at 95 °C, annealing for 30 s at 55 °C and elongation for 45 s at 72 °C, the last step being extension at 72 °C for 10 minutes.

Table S4. Data size of metagenome sequences.

Sample	Raw reads (GB)	Clean reads (GB)
P1_1	11.82	11.40
P1_2	12.01	11.50
P1_3	11.41	10.98
P1_4	11.20	10.80
P5_1	13.09	12.58
P5_2	12.57	12.17
P5_3	10.58	10.28
P5_4	12.00	11.47
Total	94.67	91.18

Table S5. Two-way ANOVA investigating the factors affecting the belowground photosynthesized C allocation, chemodiversity and potential transformations of peanut rhizodeposits.

	Sum of squares	*F*	*P*
Belowground C allocation			
Growth stage	1198.110	20.960	<0.001
Monoculture duration	1247.300	10.910	<0.001
Growth stage * Monoculture duration	671.610	5.876	0.011
Chemodiversity of peanut rhizodeposits			
Growth stage	6.471	7.663	0.013
Monoculture duration	1.157	6.852	0.006
Growth stage * Monoculture duration	4.30e5	0.255	0.778
Potential transformations of peanut rhizodeposits			
Growth stage	1.359	22.130	<0.001
Monoculture duration	1.089	17.740	0.001
Growth stage * Monoculture duration	3.256	0.530	0.48

Table S6. Correlations between belowground C allocation and plant and soil physicochemical properties. The values are Spearman correlation coefficients. *, **, and *** represent significant correlation under $P < 0.05$, 0.01, and 0.001, respectively.

	Belowground C allocation	
	W6	W10
Plant biomass		
Total plant biomass	0.671*	0.311
Aboveground plant biomass	0.657*	-0.001
Belowground plant biomass	0.743**	0.685*
Soil physicochemical properties		
pH	0.872***	0.256
SOC	-0.774**	-0.027
TN	-0.763**	-0.381
TP	-0.173	-0.193
TK	-0.407	-0.706*
AN	-0.764**	-0.296
AP	0.739**	0.093
AK	-0.603*	0.405

Table S7 Correlations between soil physicochemical properties and rhizodeposits chemodiversity. The values are Spearman correlation coefficients. *, **, and *** represent significant correlation under $P < 0.05$, 0.01, and 0.001, respectively.

	Rhizodeposits chemodiversity	
	W6	W10
pH	0.653*	0.362
SOC	-0.545	-0.547
TN	-0.684*	-0.495
TP	-0.517	-0.081
TK	-0.495	-0.077
AN	-0.539	-0.747*
AP	0.617*	0.239
AK	-0.401	0.31

Table S8. Two-way PERMANOVA investigating the factors affecting the composition of rhizodeposits and structure of the active bacterial community in the peanut rhizosphere.

	Sum of squares	*F*	*P*
Composition of peanut rhizodeposits			
Growth stage	2.21e7	17.991	<0.001
Monoculture duration	1.53e7	6.246	<0.001
Growth stage * Monoculture duration	5.61E6	2.287	0.041
Composition of active bacterial community			
Growth stage	0.498	3.672	0.004
Monoculture duration	1.086	8.014	<0.001
Growth stage * Monoculture duration	0.358	2.64	0.021

Supplementary references

1. Zhran M, Ge TD, Tong YY, Zhu ZK, Deng YW, Fahmy A, Chen M, Lynn TM, Wu JS, Gunina A. Effect of N and P fertilization on the allocation and fixation of photosynthesized carbon in paddy soil. Ecosyst Health Sust. 2021;7.
2. Stubner S. Enumeration of 16S rDNA of Desulfotomaculum lineage 1 in rice field soil by real-time PCR with SybrGreen (TM) detection. J Microbiol Meth. 2002;50:155-164.
3. Bolyen E, Rideout JR, Dillon MR, Bokulich N, Abnet CC, Al-Ghalith GA, Alexander H, Alm EJ, Arumugam M, Asnicar F *et al*. Reproducible, interactive, scalable and extensible microbiome data science using QIIME 2. Nat Biotechnol. 2019;37:852-857.
4. Callahan BJ, McMurdie PJ, Rosen MJ, Han AW, Johnson AJA, Holmes SP. DADA2: High-resolution sample inference from Illumina amplicon data. Nat Methods. 2016;13:581-+.
5. McKnight DT, Huerlimann R, Bower DS, Schwarzkopf L, Alford RA, Zenger KR. Methods for normalizing microbiome data: An ecological perspective. Methods Ecol Evol. 2019;10:389-400.
6. Wang Q, Garrity GM, Tiedje JM, Cole JR. Naive Bayesian classifier for rapid assignment of rRNA sequences into the new bacterial taxonomy. Appl Environ Microb. 2007;73:5261-5267.
7. Price MN, Dehal PS, Arkin AP. FastTree: Computing Large Minimum Evolution Trees with Profiles instead of a Distance Matrix. Mol Biol Evol.

2009;26:1641-1650.

8. Caporaso JG, Bittinger K, Bushman FD, DeSantis TZ, Andersen GL, Knight R. PyNAST: a flexible tool for aligning sequences to a template alignment. Bioinformatics. 2010;26:266-267.

9. Huang Y, Niu BF, Gao Y, Fu LM, Li WZ. CD-HIT Suite: a web server for clustering and comparing biological sequences. Bioinformatics. 2010;26:680-682.

10. Qin JJ, Li YR, Cai ZM, Li SH, Zhu JF, Zhang F, Liang SS, Zhang WW, Guan YL, Shen DQ *et al*. A metagenome-wide association study of gut microbiota in type 2 diabetes. Nature. 2012;490:55-60.

11. Danczak RE, Chu RK, Fansler SJ, Goldman AE, Graham EB, Tfaily MM, Toyoda J, Stegen JC. Using metacommunity ecology to understand environmental metabolomes. Nat Commun. 2020;11.

12. Dini-Andreote F, Stegen JC, van Elsas JD, Salles JF. Disentangling mechanisms that mediate the balance between stochastic and deterministic processes in microbial succession. P Natl Acad Sci USA. 2015;112:E1326-E1332.
